# Prognostic Impact of Bone Metastasis on Survival Outcomes in Patients with Metastatic Renal Cell Carcinoma Treated by First Line Tyrosine Kinase Inhibitors: A Propensity-Score Matching Analysis

**DOI:** 10.7150/jca.48062

**Published:** 2020-10-18

**Authors:** Minyong Kang, Joongwon Choi, Jungyu Kim, Hyun Hwan Sung, Hwang Gyun Jeon, Byong Chang Jeong, Seong Soo Jeon, Hyun Moo Lee, Se Hoon Park, Cheryn Song, Seong Il Seo

**Affiliations:** 1 Department of Urology, Samsung Medical Center, Sungkyunkwan University School of Medicine, Seoul, South Korea; 2 Department of Health Sciences and Technology, SAIHST, Sungkyunkwan University, Seoul, South Korea; 3 Department of Digital Health, SAIHST, Sungkyunkwan University, Seoul, South Korea; 4 Department of Urology, Veterans Health Service (VHS) Medical Center, Seoul, South Korea; 5 Division of Hematology-Oncology, Department of Medicine, Samsung Medical Center, Sungkyunkwan University School of Medicine, Seoul, South Korea; 6 Department of Urology, Asan Medical Center, University of Ulsan College of Medicine, Seoul, South Korea

**Keywords:** metastatic renal cell carcinoma, tyrosine kinase inhibitors, bone metastasis, survival, propensity-score matching analysis

## Abstract

**Purpose:** To investigate the effect of bone metastasis (BM) on survival outcomes in patients with metastatic renal cell carcinoma (mRCC) treated with first-line tyrosine kinase inhibitors (TKI) by performing propensity-score matching (PSM) analysis.

**Materials & Methods:** We retrospectively reviewed 1,151 patients with mRCC who were treated with first-line TKI from December 2006 to September 2016. After excluding 135 patients, 1,016 patients with mRCC were finally analyzed. The primary and secondary end points were overall survival (OS) and progression-free survival (PFS), respectively. After 1:1 PSM analysis, survival outcomes were compared between patients with BM (n*=*237) and without BM (n*=*237). Multivariate Cox regression analysis was used to determine predictors of survival.

**Results:** Among 1,016 total patients, 27.5% (n*=*279) had BM. Before PSM, patients with BM had worse OS outcomes than those without BM. Even after PSM, OS was significantly poorer in patients with BM compared to those without BM. Of note, the presence of BM was identified as an independent predictor of OS (HR=1.36), in addition to prior nephrectomy, sarcomatoid differentiation, and IMDC risk group. However, there were no differences in PFS according to the presence of BM after PSM. In the subgroup analysis, only intermediate IMDC risk group showed significant differences in OS according to the presence of BM.

**Conclusion:** Based on PSM analysis, the presence of BM negatively affected OS outcomes in patients with mRCC treated with first-line TKI, particularly in the IMDC intermediate risk group.

## Introduction

Renal cell carcinoma (RCC) is usually diagnosed as an incidental finding due to advanced imaging methods. However, approximately 15 to 30% of patients have metastatic disease at initial diagnosis 1. The five-year survival rate of patients with metastatic RCC (mRCC) is only 10% 2. Lung is the most common metastatic site, and bone is the second most common metastatic site, accounting for 30% of all metastatic lesions 3. Because bone metastasis (BM) is mainly an osteolytic process in RCC, it compromises bone integrity and frequently causes skeletal-related events (SRE) such as pain, pathologic fracture, spinal cord compression, and hypercalcemia 4. Therefore, the presence of BM is significantly associated with disease prognosis and quality of life in patients with mRCC 4.

While many studies have revealed that the presence of BM is associated with poor prognostic factors, some patients with mRCC and BM showed longer survival 5-12. In most cases, the predicted prognosis is poor, and only palliative treatments are considered in patients with BM. Furthermore, BM is usually associated with more aggressive pathologic features, such as a higher percentage of nuclear grade 4 tumor and more distant metastases at initial diagnosis 13. However, prolonged survival in patients with mRCC and BM is not rare, too. Actually, recent studies showed that median OS following initial diagnosis of BM in patients with mRCC ranged up to 40 months 9, 11, 12. Therefore, current knowledge on the prognostic impact of BM in patients with mRCC remains controversial.

Here, we examined the impact of BM on survival outcomes in more than 1,000 patients with mRCC who underwent first-line targeted therapy by performing propensity-score matching (PSM) analysis.

## Patients and Methods

### Study population

A total of 1,151 cases of mRCC in patients treated with first-line tyrosine kinase inhibitor (TKI) at Samsung Medical Center and Asan Medical Center from December 2006 to September 2016 were retrospectively collected. The electronic medical records of these patients were reviewed. All patients had measurable metastatic lesions on either computed-tomography (CT) scan or magnetic resonance imaging (MRI) and bone scan. After 135 patients were excluded from the analysis due to insufficient clinical data regarding metastatic lesions or choice of therapeutic options, 1,016 patients with mRCC were finally included in the analysis.

### Study design

Clinicopathological variables including age at initial systemic treatment, sex, type of metastasis (synchronous vs. metachronous), presence of bone metastasis, number of metastases, clinical T and N stage, histologic subtype, Fuhrman nuclear grade, history of prior nephrectomy, presence of sarcomatoid differentiation, type of first-line TKI, and the International Metastatic RCC Database Consortium (IMDC) risk classification were assessed. The primary end point was overall survival (OS), and the secondary endpoint was progression-free survival (PFS).

To reduce the selection bias of this retrospective study, PSM was performed depending on the presence of BM. Propensity-scores were calculated using a logistic regression model including the following variables: age, sex, metastasis type, IMDC risk classification, prior nephrectomy, single or multiple metastasis, histology type, Fuhrman nuclear grade, presence of sarcomatoid component, and type of TKI. Thereafter, the nearest neighbor 1:1 matching method was adopted without replacement. Adequate balance was achieved after PSM, as shown in Supplementary Figure S1. Each of the 237 patients was assigned 1:1, and the results before and after matching were compared to ensure consistency of the results. Additionally, subgroup analysis of OS estimates was also carried out according to IMDC classification after PSM.

### Statistical analysis

The statistics and data center of Samsung Medical Center supported all statistical analysis in the present study. All data are presented as numbers with percentages except age (median with interquartile range). The Chi-square test was used to analyze categorical variables, and the student *t*-test was used to compare age at baseline demographics. Survival curves were calculated by the Kaplan-Meier method, and statistical significance was determined by the log-rank test. OS was measured from the date of targeted therapy initiation to the date of death due to any cause. PFS was measured from the date of targeted therapy initiation to the date of progression, treatment cessation, or any cause of death. Multivariable Cox analysis was performed to identify independent risk factors for each survival endpoint. All statistical analysis including PSM was executed using SAS version 9.4 (SAS Institute, Cary, NC) and survival graphs were plotted with Medcalc version 14.8.1 (Medcalc software, Acacialaan, Ostend, Belgium).

## Results

The clinicopathologic characteristics of the total 1,016 patients are demonstrated in Table S1. Among these, 27.5% (n*=*279) patients had BM with or without other distant metastases. Of these patients, 23.0%, 62.0%, and 14.0% of patients had favorable, intermediate, and poor IMDC risk classification, respectively. Before PSM, more patients with BM had synchronous metastases (65.6% versus 51.3%, *P*<0.001) and multiple metastases (75.6% versus 48.8%, *P*<0.001) than patients without BM. In addition, patients with BM showed a higher proportion of poor IMDC risk classification than those without BM (21.1% versus 11.3%, *P*<0.001). After performing PSM, there were no significant differences in clinicopathologic parameters of patients with and without BM (Table 1).

**Figure 1:**
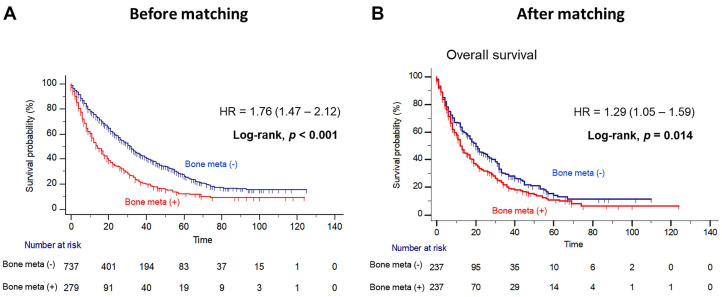
Kaplan Meier analysis estimating overall survivals (**A**) before and (**B**) after propensity-score matching according to presence of bone metastasis in patients with metastatic renal cell carcinoma.

**Figure 2:**
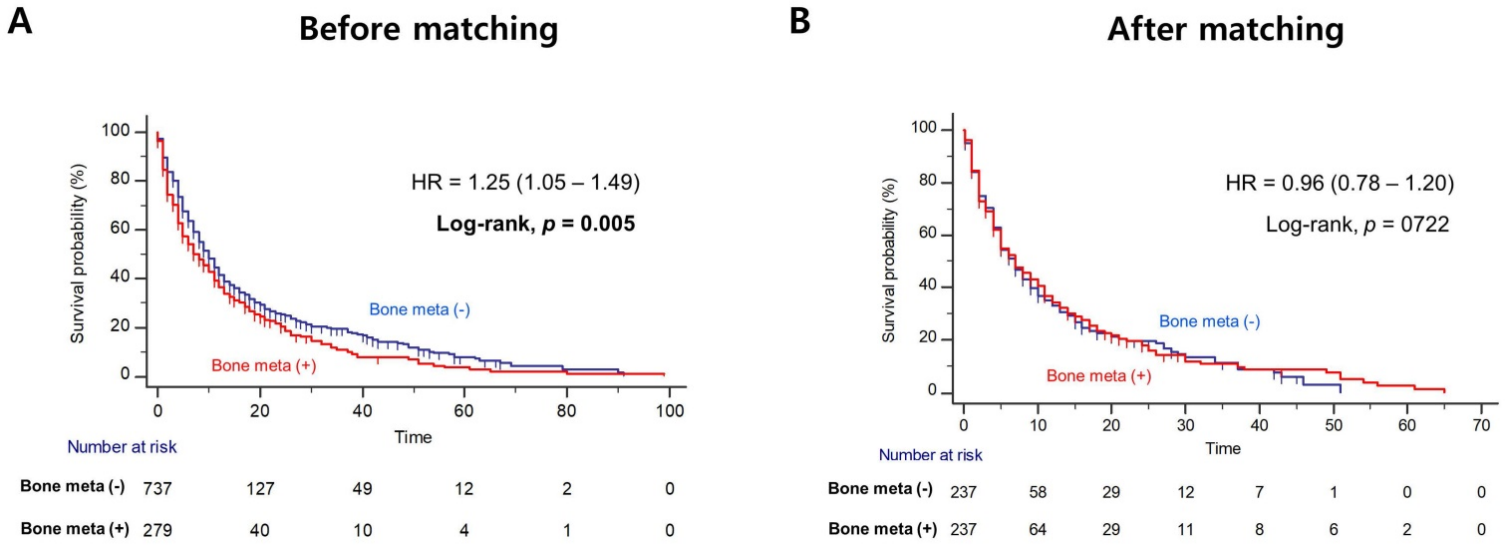
Kaplan Meier analysis estimating progression-free survivals (**A**) before and (**B**) after propensity-score matching according to presence of bone metastasis in patients with metastatic renal cell carcinoma.

Patients with BM showed worse OS outcome compared to those without BM (median OS = 14.0 versus 31.0 months; Log-rank, *P*<0.001), before PSM (Figure 1A). Notably, patients with BM had significantly poorer OS outcome than patients without BM (median OS = 12.0 versus 20.0 months, Log-rank *P=*0.014), after PSM (Figure 1B). In multivariate Cox regression analysis, the presence of BM was identified as a poor predictor of OS outcome in patients with mRCC treated with first-line TKI (HR = 1.36, 95% CI = 1.07 - 1.72). Additionally, prior nephrectomy (HR = 0.61, 95% CI = 0.47 - 0.79), sarcomatoid component (HR = 1.72, 95% CI = 1.21 - 2.43) and poor IMDC risk group (HR = 2.34, 95% CI = 1.56 - 3.48) were remained as predictors of OS outcome (Table 2). However, there was no difference in PFS according to the presence of BM after PSM (Figure 2).

**Figure 3:**
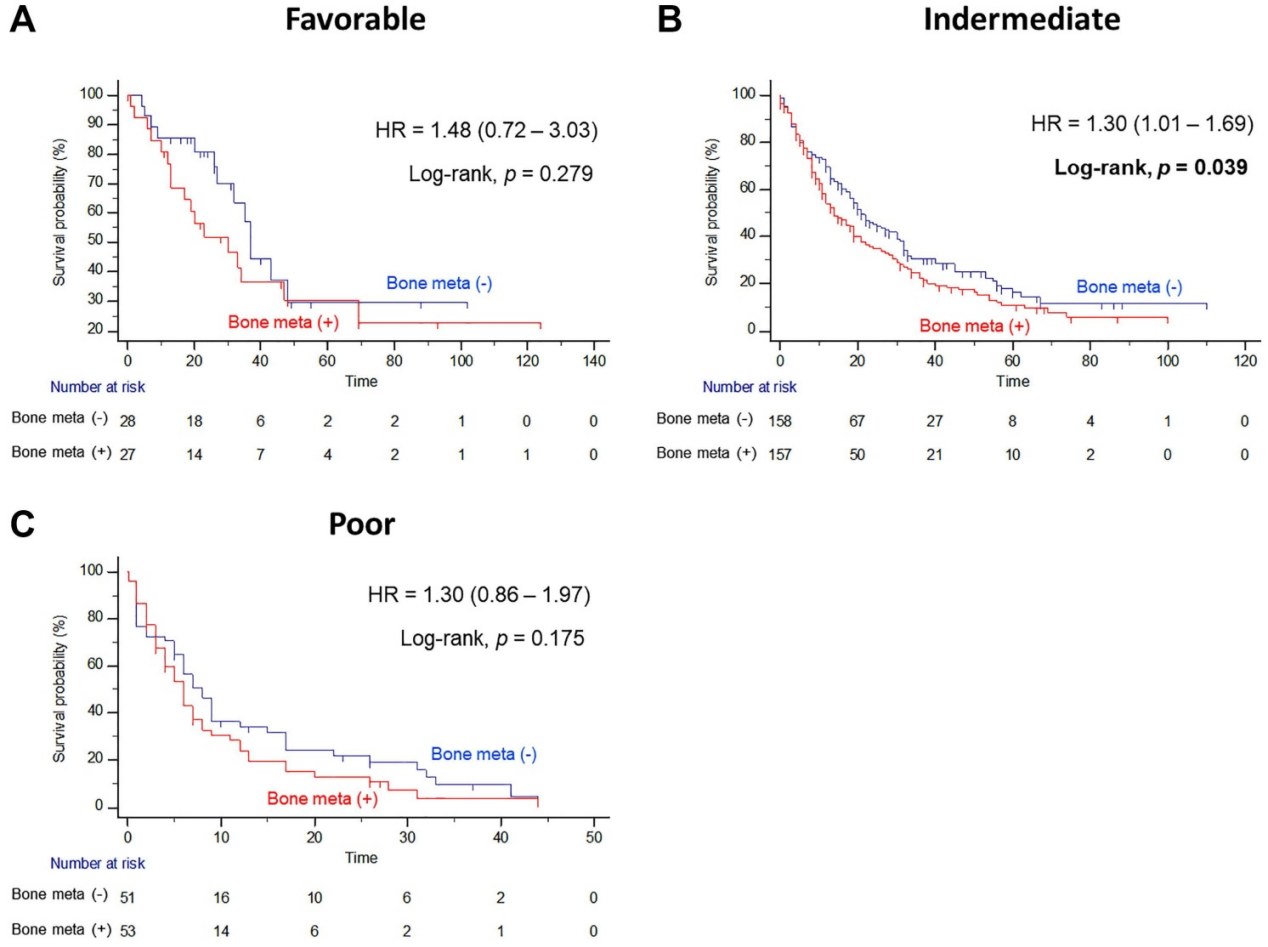
Kaplan Meier analysis estimating overall survivals after propensity-score matching according to presence of bone metastasis in (A) favorable, (B) intermediate, and (C) poor International Metastatic RCC Database Consortium risk groups in patients with metastatic renal cell carcinoma.

In subgroup analysis, the prognostic significance of BM was examined according to IMDC risk classification. Interestingly, the presence of BM resulted in poor OS outcomes only in the intermediate risk group after PSM (Figure 3B). The median OS of patients with BM was seven months shorter than the median OS of patients without BM (14.0 versus 21.0 months, Log-rank *P=*0.039), and the presence of BM significantly increased the risk of any cause of death (HR = 1.30, 95% CI = 1.01 - 1.69). However, there were no statistically significant differences in OS estimates between patients with and without BM both in favorable and poor IMDC risk groups (Figure 3A and 3C, respectively).

The prognostic impact of BM in patients with solitary metastasis was also analyzed. Prior to PSM, patients with solitary BM showed worse OS outcome than those with solitary metastases on other sites (median OS = 23.0 versus 38.0 months; Log-rank, *P*<0.001) (Supplementary Figure S2A). After performing PSM, patients with solitary BM also had significantly worse OS outcomes than those with other solitary metastases (median OS = 11.0 versus 23.0 months; Log-rank, *P=*0.035) (Supplementary Figure S2B). In subgroup analysis among cases of solitary metastasis, the negative impact of BM on survival outcome was only observed in the intermediate IMDC risk population (Supplementary Figure S3).

## Discussion

The reason for poor survival outcomes in patients with BM has been suggested as follows: Interactions between cancer cells and the tumor microenvironment, particularly the bone microenvironment, can result in bone destruction and rapid tumor growth 4. In addition, TKI has limited distribution to bone, compromising the anti-tumor effects on BM 14, 15. Despite several studies revealing that BM is associated with higher risk of morbidity and shorter survival in patients with mRCC, study population heterogeneity was a critical drawback 5-12. Moreover, there are no standard therapeutic guidelines or prognostic systems for patients with BM. Thus, decision-making for these patients is largely determined empirically 16. The prognostic impact of BM on survival outcomes in patients with mRCC still remains an open question. Of note, our study adds new evidence supporting that the presence of BM is associated with significant negative impact on OS, but not PFS.

The proportion of BM in our series was approximately 27%, which is similar to other reports indicating BM metastases as comprising one third of all metastatic sites. However, median OS was 14 months and 12 months before and after PSM, respectively, which was shorter than in other studies. Ruatta et al 9 analyzed 300 patients with BM among 1,750 patients with mRCC and showed that median OS was 23.3 months. Particularly, they highlighted that patients with a solitary bone lesion had a longer survival than patients with multiple BMs (27.7, 18.2 and 9.2 months in patients with one, two to five and more than 5 BMs, respectively, *P*<0.0001) 9. Although we also found that patients with solitary BM had longer median OS than those with concomitant BM (23 versus 14 months), the negative impact of BM was more profound in our series. This discrepancy can be explained by the heterogeneity of study population. Because we could not evaluate the distribution of the number of BM, patients may have various degree of tumor burden. This type of heterogeneity can influence the survival outcomes.

Interestingly, our data showed that the prognostic impact of BM was only significant in the population with intermediate IMDC risk classification. In this subgroup, median OS of patients with BM was 7 months shorter than those without BM (14.0 versus 21.0 months), and the presence of BM significantly increased the risk of any cause of death (HR = 1.30). Conversely, there were no statistically significant differences in OS estimates for patients with and without BM either in favorable or poor IMDC risk groups, regardless of PSM. McKay et al 17 reported that favorable and intermediate risk groups with BM had shorter OS and time to treatment failure. The authors showed that the presence of BM was an independent predictor for poor OS outcome when classified by IMDC risk criteria. In a study by Ruatta and colleagues, the MSKCC risk score was also associated with OS (HR = 0.50; 95% CI=0.38 - 0.67). In a study by Kalra et al 18, higher MSKCC risk score was an independent predictor of shorter OS in data from 375 patients with mRCC and BM (HR = 1.38; 95% CI, 1.02-1.91). Because the number of cases in a favorable and poor risk group is too low in our study, we should consider that the impact of BM on the survival outcomes could be underestimated in these population.

Other key finding of the present study is that patients with solitary BM had significantly worse OS outcomes than those with other solitary metastases. This result has various possible explanations, including different metastatic burden, different tumor biology, and tumor heterogeneity. Both the temporal and spatial patterns of distant metastasis are highly variable in patients with RCC 19. Metastatic patterns vary from indolent, step-wise spreading of single or oligometastatic sites with prolonged periods of latency between initial and subsequent events to highly aggressive early and systemic dissemination within months after surgery with curative intent 19. The local interplay between cancer cells and the bone environment with BM may contribute to progression of skeletal metastasis 4. Particularly, osteoclasts play a key role in the bone destruction of metastatic osteolytic lesions 20. Activated osteoclasts destroy bone integrity and release various cytokines and growth factors, including bone morphogenetic proteins, insulin-like growth factor, and transforming-growth factor-ß, which stimulate tumor cell proliferation 20, 21.

We acknowledge several limitations of our study. First, our data were retrospectively collected and reviewed. Despite the consecutive enrollment of patients, there is potential risk of selection and misclassification bias. Second, there were no data about the effects of palliative local treatments, such as metastasectomy and SBRT (stereotactic body radiation therapy), as well as the use of bone-targeting agents, such as bisphosphonates and denosumab (the receptor activator of nuclear factor kappa-B ligand). Actually, metastasis-directed therapy can be helpful for the patients with solitary or oligometastatic BMs. In the study by Sun and colleagues, patients treated by metastasectomy had a better survival outcome compared to those who did not underwent metastasectomy 22. In addition, SBRT has been reported to be safe and feasible in patients with oligometastasis, particularly with high local control rate more than 90% 23. Third, our data did not provide information regarding accurate tumor burden and involved sites of BM. As previously described, the study by Ruatta et al 9 showed that patients with a solitary BM had a better prognosis compared to those with multiple BMs. Furthermore, the location of BMs had a prognostic impact on survival in patients with BM, and patients with long bone metastasis had a longer survival than patients with spinal column or sacrum metastases 9. Finally, although cabozantinib treatment showed better clinical benefits in mRCC patients with BM after previous VEGFR targeted therapy 24, we could not assess the effects of cabozantinib in the current study because the study was performed in patients prior to FDA approval of cabozantinib in our country. Therefore, we only focused on the population who received first-line TKI therapy in this study.

## Conclusions

In sum, our study showed that the presence of BM negatively affected OS outcome in patients with mRCC treated with first line TKI, particularly in the IMDC intermediate risk group, by performing PSM analysis. Even in patients with solitary metastasis, those with BM had worse OS compared to other solitary metastases. Increased understanding of the prognostic impact of BM is essential to provide better personalized therapeutic decisions in patients with mRCC.

## Supplementary Material

Supplementary figures and table.

## Figures and Tables

**Table 1:** Comparison of baseline demographics according to presence of bone metastasis (BM) in patients with metastatic renal cell carcinoma treated by first-line targeted therapy after propensity-score matching

Variables	No BM	BM	Total	*P-*value
No.	237 (50.0%)	237 (50.0%)	474 (100%)	
Age	59.0 (51.0-68.0)	59.0 (51.0-67.0)	59.0 (51.0-68.0)	0.610
Sex				0.447
Male	177 (74.7%)	184 (77.6%)	361 (76.2%)	
Female	60 (25.3%)	53 (22.4%)	113 (23.8%)	
Metastasis type				0.913
Synchronous	171 (72.2%)	172 (72.6%)	343 (72.4%)	
Metachronous	66 (27.9%)	65 (27.4%)	131 (27.6%)	
IMDC risk classification				0.992
Favorable	28 (11.8%)	27 (11.4%)	55 (11.6%)	
Intermediate	158 (66.7%)	157 (66.2%)	315 (66.5%)	
Poor	51 (21.5%)	53 (22.4%)	104 (21.9%)	
Prior nephrectomy	151 (63.7%)	151 (63.7%)	302 (63.7%)	0.554
Number of metastases				0.896
Single	60 (25.3%)	59 (24.9%)	119 (25.1%)	
Multiple (≥ 2)	177 (74.7%)	178 (75.1%)	355 (74.9%)	
Histology				0.409
Clear cell	114 (48.1%)	100 (42.2%)	214 (45.1%)	
Non-clear cell	15 (6.3%)	23 (9.7%)	38 (8.0%)	
Unknown	108 (45.6%)	114 (48.1%)	222 (46.8%)	
Fuhrman nuclear grade				0.972
Low	15 (6.3%)	17 (7.2%)	32 (6.8%)	
High	59 (24.9%)	57 (24.1%)	116 (24.5%)	
Unknown	163 (68.8%)	163 (68.8%)	326 (68.8%)	
Sarcomatoid component	22 (9.3%)	20 (8.4%)	42 (8.9%)	0.731
First line treatment				0.999
Sunitinib	136 (63.6%)	139 (65.0%)	275 (64.3%)	
Sorafenib	26 (12.2%)	23 (10.8%)	49 (11.5%)	
Pazopanib	49 (22.9%)	47 (22.0%)	(22.4%)	
Others	26 (1.3%)	28 (2.2%)	54 (1.8%)	

**Table 2:** Multivariate Cox regression analysis identifying predictors of overall survival in patients with metastatic renal cell carcinoma treated by first-line targeted therapy

	Multivariate Cox regression				
		Hazard Ratio	95% Hazard Ratio	Confidence Limits	*P-*value
**Bone metastasis**	No	Ref			
	**Yes**	1.36*	**1.072**	**1.722**	**0.011**
Metastasis type	Synchronous	Ref			
	Metachronous	0.86	0.652	1.138	0.294
**Prior nephrectomy**	No	Ref			
	**Yes**	0.61*	**0.472**	**0.795**	**< 0.001**
**Sarcomatoid component**	No	Ref			
	**Yes**	1.72*	**1.215**	**2.437**	**0.002**
**IMDC risk classification**	Favorable	Ref			
	Intermediate	1.35	0.989	1.840	0.059
	**Poor**	2.34*	**1.564**	**3.486**	**<0.001**
Metastasis number	Single	Ref			
	Multiple	1.10	0.831	1.451	0.510

^*^P<0.05
