# Supplementary material for: Prognostic Impact of Bone Metastasis on Survival Outcomes in Patients with Metastatic Renal Cell Carcinoma Treated by First Line Tyrosine Kinase Inhibitors: A Propensity-Score Matching Analysis
Source: J Cancer. 2020 Oct 18;11(24):7202–8. doi: 10.7150/jca.48062 (PMC7646162; doi:10.7150/jca.48062)
Supplement: Supplementary file 1 — Supplementary figures and table. [file jcav11p7202s1.pdf]

Supplementary Figure S1. Distribution balance bafore and after propensity-score matching

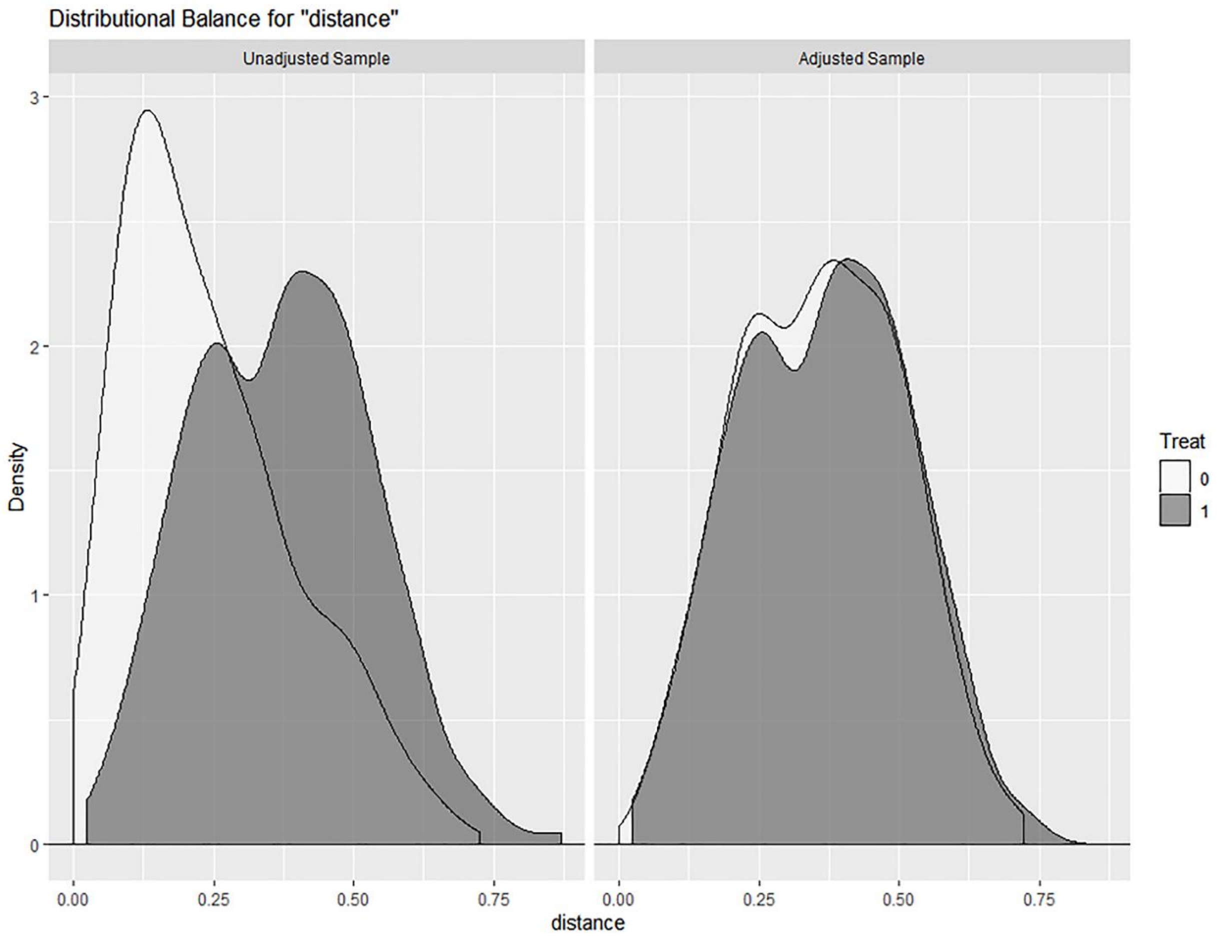

Supplementary Figure S2. Kaplan Meier analysis estimating overall survivals (**A**) before and (**B**) after propensity-score matching according to presence of either bone metastasis or other solitary metastases in patients with metastatic renal cell carcinoma.

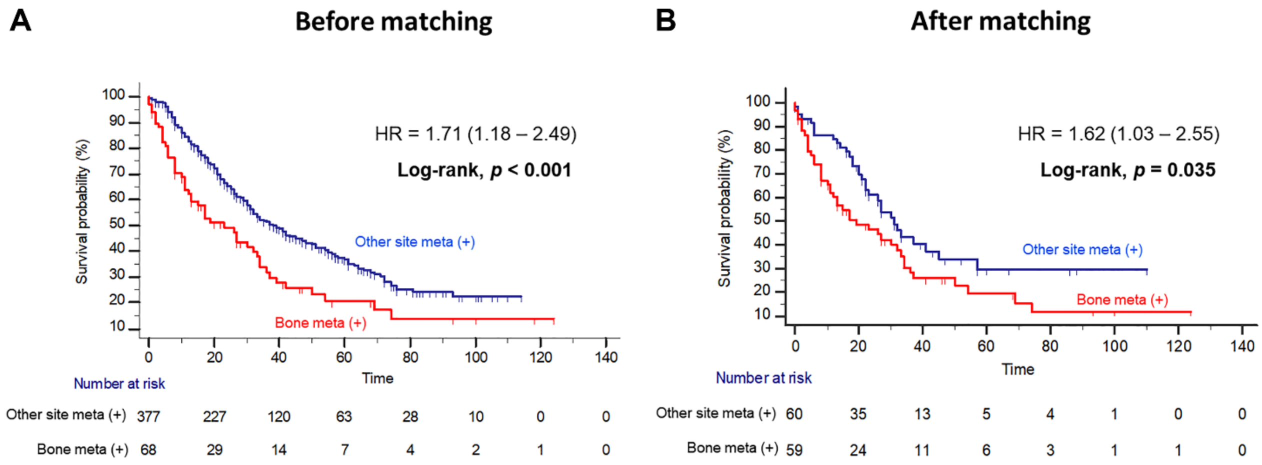

Supplementary Figure S3. Kaplan Meier analysis estimating overall survivals after propensity-score matching according to presence of either bone metastasis or other solitary metastases in (A) favorable, (B) intermediate, and (C) poor IMDC risk groups in patients with metastatic renal cell carcinoma.

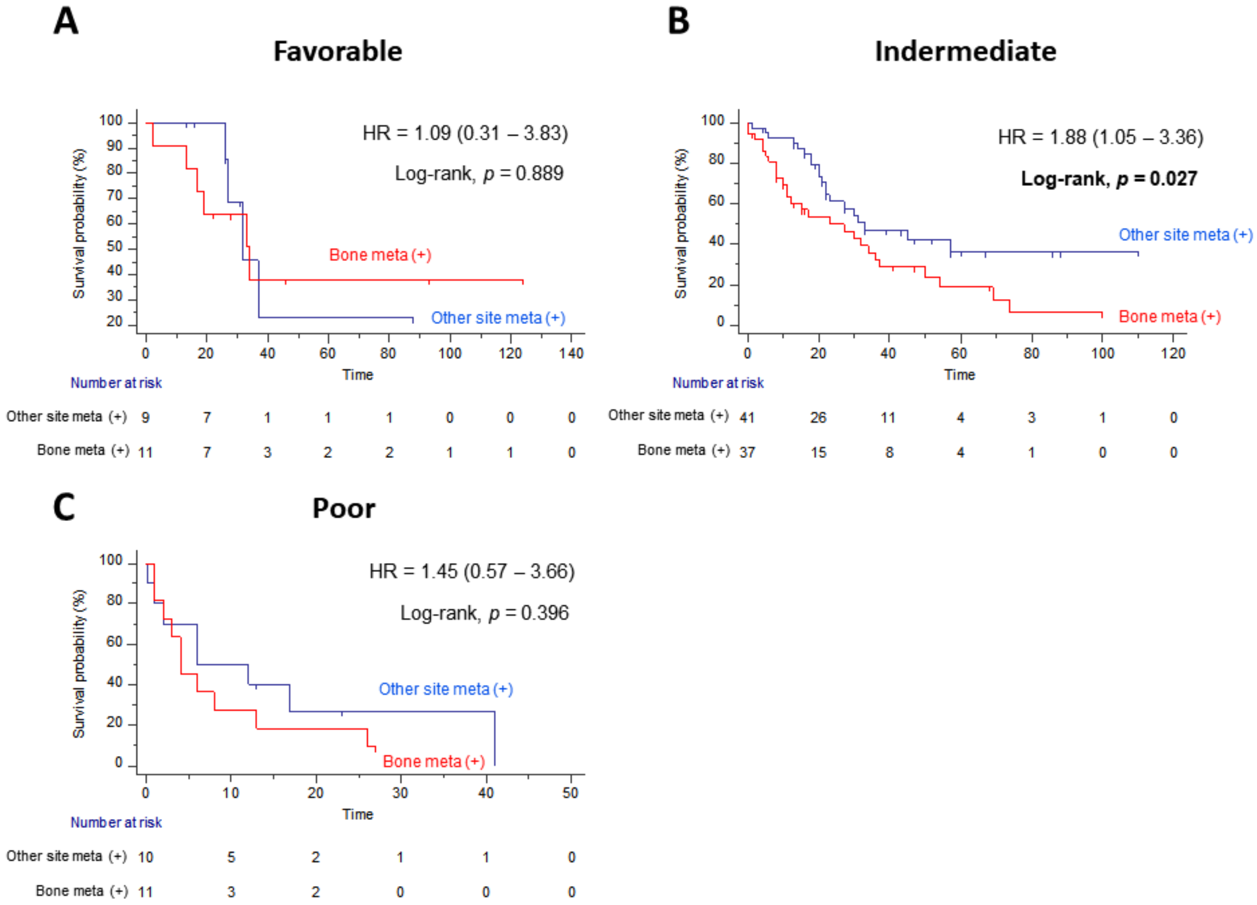

Table S1. Comparison of baseline demographics according to presence of bone metastasis (BM) in patients with metastatic renal cell carcinoma treated by first-line targeted therapy before propensity-score matching

**Variables**	**No BM**	**BM**	**Total**	***P*-value**
No.	737 (72.5%)	279 (27.5%)	1016 (100%)	
Age	60.0 (52.0–68.0)	58.0 (50.0–67.0)	60.0 (51.0–67.0)	0.153
Sex				0.959
Male	565 (76.7%)	215 (77.1%)	780 (76.8%)	
Female	172 (23.3%)	64 (22.9%)	236 (23.2%)	
Metastasis type				< 0.001
Synchronous	378 (51.3%)	183 (65.6%)	561 (55.2%)	
Metachronous	359 (48.7%)	96 (34.4%)	455 (44.8%)	
IMDC risk classification				< 0.001
Favorable	194 (26.3%)	40 (14.3%)	234 (23.0%)	
Intermediate	451 (61.2%)	179 (64.2%)	630 (62.0%)	
Poor	83 (11.3%)	59 (21.1%)	142 (14.0%)	
Unknown	9 (1.2%)	1 (0.4%)	10 (1.0%)	
Clinical T stage				< 0.001
≤ cT2	118 (16.0%)	86 (30.8%)	204 (20.1%)	
≥ cT3	238 (32.3%)	72 (25.8%)	310 (30.5%)	
Unknown	381 (51.7%)	121 (43.4%)	502 (49.4%)	
Clinical N stage				< 0.001
cN0	201 (27.3%)	123 (44.1%)	324 (31.9%)	
cN1	167 (22.7%)	57 (20.4%)	224 (22.0%)	
Unknown	369 (50.1%)	99 (35.5%)	468 (46.1%)	
Type of first-line therapy				0.004
Sunitinib	416 (56.4%)	163 (58.4%)	579 (57.0%)	
Sorafenib	96 (13.0%)	27 (9.7%)	123 (12.1%)	
Pazopanib	173 (23.5%)	53 (19.0%)	226 (22.2%)	
Others	52 (7.0%)	36 (12.9%)	88 (8.7%)	
Number of metastases				< 0.001
Single	377 (51.2%)	68 (24.4%)	445 (43.8%)	
Multiple (≥ 2)	360 (48.8%)	211 (75.6%)	571 (56.2%)	
